# Microbial Science Research in the Post-COVID Environment

**DOI:** 10.1128/mBio.01116-21

**Published:** 2021-05-28

**Authors:** Nguyen K. Nguyen, Carey-Ann D. Burnham, Arturo Casadevall, Mary K. Estes, Rebecca V. Ferrell, Suzanne M. J. Fleiszig

**Affiliations:** a American Academy of Microbiology, Washington, DC, USA; b Washington University School of Medicine, Pathology & Immunology, St. Louis, Missouri, USA; c Johns Hopkins Bloomberg School of Public Health, Baltimore, Maryland, USA; d Baylor College of Medicine, Molecular Virology and Microbiology, Houston, Texas, USA; e Metropolitan State University of Denver, Denver, Colorado, USA; f University of California, Berkeley, School of Optometry, Berkeley, California, USA; g Participants listed in Acknowledgments.; University of Michigan—Ann Arbor

**Keywords:** big data, collaboration, pandemic response, coronavirus, public health

## Abstract

Since early 2020, the world has witnessed the unprecedented accomplishments of the scientific community in the fight against the coronavirus disease 2019 (COVID-19) pandemic. In the meantime, we also learned valuable lessons and recognized the challenges that hindered our successes. In this article, we synthesize the ideas discussed at the ASM Virtual Symposium: Microbial Science Research in the Post-COVID Environment on 10 November 2020. We propose three new approaches that microbiology researchers can embrace to overcome these challenges. Moreover, we suggest broad systematic changes to focus on social impacts, teamwork, and diversity, equity, and inclusion. We believe these values are needed to prepare the microbial science research community for future opportunities and challenges.

## PERSPECTIVE

Though not completely unexpected, the coronavirus disease 2019 (COVID-19) pandemic caught the scientific and medical community and the rest of the world off guard. Countries struggled to contain the outbreak. Most failed. Globally, at the time of preparing this article, the pandemic has already taken over 2 million lives and cost the world economy 8 to 16 trillion U.S. dollars (1). During the same period, health care providers and scientists across the globe quickly shifted their efforts and joined the quest to fight against the severe acute respiratory syndrome coronavirus 2 (SARS-CoV-2) virus. The stakes were high; the pressure was immense.

In a short period, the progress made by the scientific community to fight the pandemic has been unprecedented. By January of 2021, after a single year of the pandemic, more than 93,000 papers were published, and 319 treatments and 242 vaccine candidates were in the pipeline (2, 3). In the United States, the FDA has granted emergency use authorization (EUA) on over 200 molecular diagnostics, 69 serologic assays, and 13 antigen tests (4). By March of 2021, three COVID-19 vaccines from Pfizer-BioNTech, Moderna, and Johnson & Johnson were approved by the FDA, and over 125 million doses have already been administered. These significant scientific achievements bring much hope and should be jointly celebrated. All these accomplishments are strong testimony that decades of investment in scientific research have resulted in important solutions when the world most needed help.

On the other hand, the COVID-19 pandemic also exposed the weaknesses of the scientific community on multiple fronts. The so-called “sandbag mindset” common in current science that emphasizes short-term and safe returns such as getting the next grants, proposing only fail-proof ideas, overvaluing publication numbers, overemphasizing manuscript authorship positions, etc., has revealed many limits when it comes to rapid innovation and saving lives. In his book *The Structure of Scientific Revolutions*, Kuhn describes this feature of modern scientific culture: “The most striking feature of the normal research problems we have just encountered is how little they aim to produce major novelties, conceptual or phenomenal.” (5). Therefore, the research community must take a closer look at the lessons learned, embark on decisive actions to fill the gaps, and diligently prepare for the next big event.

Regardless of whether the next big thing will be a novel scientific breakthrough or another humanity-threatening pandemic, moving forward, researchers—especially microbiologists—need to shift to new approaches and embrace new values in scientific partnership to thrive in the post-COVID world.

## NEW APPROACHES FOR THE POST-COVID WORLD

### Focus on the new mindset.

“Any useful idea about the future should appear to be ridiculous,” according to Jim Dator, futurist at the University of Hawaiʻi at Mānoa (6), who taught students to think broadly and consider long-term possibilities, looking up to 30 years into a future where data are not yet available. This mindset aims to open the thoughts of futurists to the possibilities that emerge from asking “what if” and considering all potential scenarios.

What if the next drug-resistant microbe emerges within the next year? What if the next pandemic occurs in 2025, or 2035, or 2045? What if climate change alters microbe compositions and results in widespread food shortage, new pathogens of humans and animals, or the resurgence of old microbial enemies? Will we be asking the same questions as we did in our last grant proposals, or will our questions perhaps be grander? Will we be using broader approaches, collaborating with those we previously had not imagined as part of the team? Arguably, this shift in mindset is the critical focal point needed for adapting to the new normal. Futuristic thinking naturally focuses on the needs and significance of the research being done, taking on risks, establishing partnerships, and building needed tools. Microbiologists need to be creative in recognizing how they can use their expertise to solve the current or next challenge.

### Focus on harvesting the power of big data and artificial intelligence to drive science.

Data have always been the heart of science. With the advancement of technology, the approaches to collect, share, analyze, and utilize data today have improved significantly compared with the last decades. The 20th century paradigm of human-generated data limits how the scientific community approaches research questions in the 21st century. Today, machines can produce and analyze a vast amount of data that a human scientist cannot easily match. While big data, machine learning, and artificial intelligence (AI) are making their way into the clinical settings to augment clinical staff in making clinical decisions, they are among the least-used tools by basic science researchers.

Because researchers are already in the business of generating, organizing, and sharing data, the good news is the hurdle for researchers to utilize big data and AI should not be too difficult to overcome. To accelerate scientific progress in the 21st century, researchers must ask the critical questions shown in Table 1.

**TABLE 1:** Critical data questions for researchers

Data issue	Critical questions for researchers
Data accessibility	How much of my data are accessible to the scientific community? Am I keeping my data in a lab notebook sitting on a shelf or are they shared with others? My next unknown collaborators may be highly interested in my data. Are my data organized in a collaboration-friendly manner?
Data format and volume	How big are my data? Are the data structured or unstructured? Are my data machine-readable? How much data do I generate monthly?
Data security	Where are my data saved? Are they properly backed up? Is the sensitive information properly protected?
Data stewardship	How well and often do I audit and organize my data besides the instances of grant and manuscript writing? Are my data harmonized and can they be combined with other data sources?
Data utilization and continuing education	Am I familiar with data science and AI or do I have a close collaborator who is? If not, how can I equip myself and/or find experts to consult?

While AI cannot replace inquisitive human minds, in time, researchers who are working with AI will likely outperform those who do not.

### Focus on expanding the research toolkit.

At the ASM Virtual Symposium Microbial Science Research in the Post-COVID Environment, clinical public health was highlighted as an important tool to help manage the pandemic. For example, the partnerships between clinical laboratories, public health officials, and environmental science professionals for contact tracing and community surveillance have resulted in better pandemic control in many communities. The CDC announced the National Wastewater Surveillance System (NWSS) as a new public health tool to understand the extent of the spread of the virus (7). Moving forward, we hope this momentum will help grow and sustain new coalitions where new thinking to accelerate research will be generated. This includes developing new approaches to communicate effectively with the public, renewing public health infrastructure to translate scientific discoveries quickly to diagnostic and treatment options in an equitable manner, and building open-source resources to equip the community to respond swiftly to the pandemic. These are all important “tools” that need to be built and assembled today for tomorrow.

Besides the typical tools, diverse skillsets are an important component of the toolkit. To be able to comprehend why the public is hesitant to accept the new COVID-19 vaccines despite their high efficacy requires knowledge in human psychology and social behaviors. Unfortunately, those skillsets have not always been emphasized in medical or graduate schools. Researchers can expand their effectiveness by equipping themselves with new skills either through learning or partnerships. A sociologist can help a vaccine researcher understand the culture and behaviors of diverse communities to design an effective strategy to overcome hesitancy and increase vaccine uptake among the underrepresented communities. The terms “microbiologists, virologists, bacteriologists, etc.” should be expanded to include all people who are working toward advancing the knowledge about microorganisms and their impacts to the society.

## A NEW VALUE SYSTEM TO BRING FORWARD SOCIAL IMPACTS

As science leads the way, the COVID-19 pandemic also brings the scientific community together in new ways. COVID and non-COVID-related researchers broke out from their scientific silos to support each other and establish interdisciplinary collaborations that have resulted in remarkably large amounts of data being generated very quickly. It also exposed the weaknesses of the current reward system that hinders these partnerships from happening in normal times. To ensure these scientific partnerships continue after the COVID-19 pandemic, a new value system will need to be established.

### A value system that emphasizes social impacts.

What is the ultimate goal of science if not to benefit society? While most scientists, in various degrees, agree with the concept, many will focus on short-term outcomes rather than long-term impacts. Others will argue that science is an expression of human curiosity and recoil at always linking scientific goals to utilitarian aims. One major reason for the hesitancy in linking science with social good is that measuring social impacts is not easy. It takes time and much more effort to implement changes that bring social impacts, while it is less work and faster to demonstrate outcomes. A strong publication record including books and high-impact journal articles, an endowed professorship, a portfolio of patents and awards, and a long list of postdoctoral and graduate students trained in the lab are all concrete items that are visible and measurable. How will one measure the effort to build a resilient public health system? A career dedicated to teaching and training underrepresented undergraduate students in research? A trusting university-community-based research partnership to study common challenges that affect the global and local communities? Social issues are complex and may take years or even decades to show an effect. But without rewards for efforts aimed at impacting society, the scientific community can easily fall back into the previous “sandbag” mindset.

### A value system that rewards groups, not just individuals.

For any complex problems, we need people from different disciplines with diverse expertise working together to address them. For COVID-19, we witnessed interdisciplinary researchers coming together to identify the viral sequence, structure, modes of transmission, host immune response, means of clinical diagnosis, and effective treatment. It is hard to pinpoint a single study or a set of work from one group that was the most significant. That is how science should be—a team effort. However, for a long time, the recognition system has focused on rewarding only individuals, undermining the values of team science. How many times do we hear people hold back data to be the first to publish? How often do we hear comments that criticize papers with a long list of authors and provide comments such as “who did the work” or questioning the contribution of each individual? Our current system encourages competition that can fuel progress during the “typical” times but can be detrimental during times of crisis. We argue that a new value system that focuses on rewarding and supporting people by the trust they build among group members and the level of support that team members provide to each other to reach shared goals will foster collaborations and success toward overcoming practical needs. These changes can break down barriers to bring researchers toward working on shared goals.

### A value system that focuses on diversity, equity, and inclusion.

Science has always benefited from the diversity of thoughts, ideas, viewpoints, expertise, and experience. Many studies have demonstrated that diverse ideas help to bring better solutions. Diversity has been viewed as a luxury rather than a necessity in the scientific value system, but we argue that at this time of social inequality, science cannot achieve excellence without diversity. Without investing in a diverse workforce, we will not reach the necessary diversity to thrive in science. There are many ways to achieve the needed diversity. For example, what if it were to become a requirement to mentor historically underrepresented students to achieve tenure at all publicly funded institutions? How about ensuring the people in the lab, the department, classroom, etc., have a voice and/or representation in major institutional decisions? Right now, these practices are rare because they are costly, messy, time-consuming, and hard to measure. We reward short-term positive returns at the cost of long-term deficits. Therefore, we argue that in the new value system, diversity, equity, and inclusion (DEI) should be a major component. When our colleagues in the business community adopt the DEI approach, the results show up in their bottom line. Organizations that embrace diversity and inclusion demonstrate more profit, less turnover, and increasing brand value. By following these practices, the scientific community will improve our productivity, attract more talent, and contribute more effectively to society.

## CONCLUSION

To promote new directions, adopt a new value system, and embrace the new mindset, we need true champions who act as trusted messengers and lead by example to bring the research community forward by applying these values. There will be challenges, questions, and opposition to these new concepts. It is important that these ideas are tested and examined. However, unless we bring forward a novel solution and change the status quo of the way we think, how we collaborate, and carry out science, we will risk stumbling in the same struggles we are dealing with during this pandemic. We are running against the next deadly microbes, and the clock is ticking. “Chance favors only the prepared mind”—Louis Pasteur.
